# Incidental Eosinophilic Chromophobe Renal Cell Carcinoma in Renal Allograft

**DOI:** 10.1155/2017/4232474

**Published:** 2017-10-09

**Authors:** Abdullah Alharbi, Maram S. Al Turki, Noura Aloudah, Khaled O. Alsaad

**Affiliations:** ^1^Department of Pathology and Laboratory Medicine, King Abdulaziz Medical City, Riyadh, Saudi Arabia; ^2^College of Medicine, King Saud bin Abdulaziz University for Health Sciences, Riyadh, Saudi Arabia

## Abstract

The incidence of renal cell carcinoma (RCC) in renal allograft in transplant recipients is 0.22–0.25%. De novo clear cell, papillary, and chromophobe RCCs and RCCs with sarcomatoid differentiation originating in renal allograft have been reported. Routine surveillance for graft tumours is not routinely practiced and these tumours are commonly asymptomatic and incidentally discovered. We describe a case of incidental, eosinophilic chromophobe RCC in a 31-year-old, long-term renal transplant male recipient, who presented with acute gastroenteritis 11 years after transplantation. The graft was nonfunctional at the time of presentation. Abdominal ultrasound and computed tomography scan demonstrated 1.8 cm well-defined, round enhancing lesion, confined to the renal allograft and suspicious for malignancy. Pathological examination of graft nephrectomy specimen showed gross, histopathological, and immunohistochemical features of eosinophilic chromophobe RCC. Fifty-five months after surgery, the patient was alive and free of malignancy. To the best of our knowledge, only five chromophobe RCCs originating in a renal allograft were previously described in English literature. We suggest that chromophobe RCC should be considered in the differential diagnosis of renal allograft mass, including eosinophilic tumours, and emphasise the importance of periodic screening of renal allograft in all renal transplant recipients.

## 1. Introduction

Patients with end stage renal disease (ESRD), particularly those with a history of chronic haemodialysis and subsequent acquired cystic kidney disease, have a high incidence of native kidney epithelial neoplasms [1]. Renal transplantation is the best treatment for patients with ESRD. Due to long-term maintenance immunosuppression therapy, transplant patients have higher incidence for benign and malignant tumours than general population [2], and cancer is a major cause of morbidity and mortality after transplantation [3]. Nonmelanoma skin, genitourinary tract malignancies, and malignant lymphoproliferative diseases are the commonest in kidney transplant recipients [4]. The risk of renal cell carcinoma (RCC) in native kidneys of transplant recipients is approximately 15–30-fold more than general population [5, 6] and the incidence of RCC in native and renal allograft in kidney transplant recipients is 1.1–1.5% and 0.22–0.25%, respectively [7]. Clear cell (conventional) and papillary RCCs are the most common histological tumour types in both native and transplanted kidney [6]. Herein, we describe a rare case of incidental de novo eosinophilic variant of chromophobe RCC (ChRCC) in a renal allograft 11 years after transplantation, discuss the histopathological differential diagnosis, and comment on cancer screening in renal transplant recipients.

## 2. Case Report

**Figure 1:**
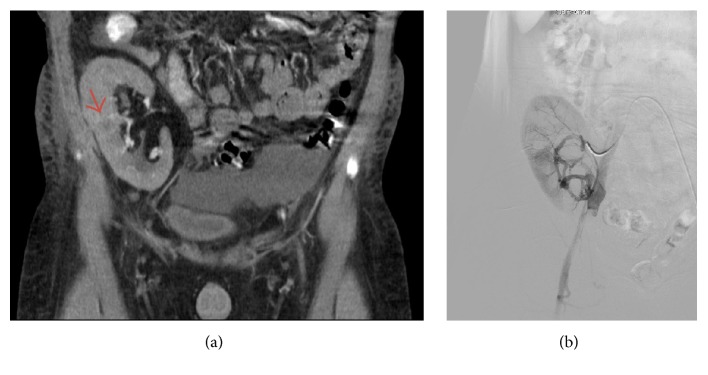
(a) Computed tomography scan coronal section demonstrates an enhancing, round lesion, suspicious for malignancy in the mid renal zone (arrow). (b) Embolization of the renal artery of the renal allograft was performed before graft nephrectomy.

A 31-year-old man underwent living nonrelated kidney transplantation at age of 16 years for ESRD secondary to biopsy-proved immune-complex mediated membranoproliferative glomerulonephritis. The patient was maintained on cyclosporine A, prednisone, and azathioprine immunosuppression regimen. His early posttransplant course was complicated with lymphocele, which was percutaneously drained and* pseudomonas aeruginosa* urinary tract infection, which was treated by meropenem. The patient was noncompliant with his immunosuppression therapy and the clinical and radiological follow-up was not optimal due to patient's poor compliance. He experienced multiple episodes of acute cellular graft rejection, which resulted in severe transplant glomerulopathy, global glomerulosclerosis, severe interstitial fibrosis, and tubular atrophy and severe chronic allograft arteriopathy. Radiological studies during the follow-up period showed no evidence of tumour neither in native kidneys nor in renal allograft. Haemodialysis was resumed 11 years after transplantation due to failed renal allograft. Four weeks after resuming haemodialysis, the patient presented to the hospital emergency with a history of watery diarrhea, vomiting, and fever associated with rigor and mild diffuse abdominal pain for 3 days. In addition, he reported an episode of gross haematuria. The patient's vital signs were as follows: body temperature 37.9°C, respiratory rate 20/min, heart rate 101/min, and blood pressure 138/93 mmHg. His oxygen saturation was 100% on room air. Physical examination revealed mild generalised abdominal and graft tenderness. The rest of the physical examination was unremarkable. Urine analysis showed white blood cells (WBC) 7/HPF, red blood cells (RBC) 1/HPF, and protein 600 mg/dl. Laboratory tests showed haemoglobin level of 143 g/l, white blood cell count 3.9 × 10^9^/L, platelet 150 × 10^9^/L, normal electrolytes, blood urea nitrogen 6.2 mmol/L, and serum creatinine 801 *μ*mol/L. Abdominal ultrasonography (US) demonstrated 14.3 cm renal allograft with well-defined, round lesion at the mid zone of the kidney, measuring 1.8 cm in maximum dimension, highly suspicious for malignancy. Both native kidneys were atrophic with no evidence of cystic changes or focal lesion. Abdominal computed tomography (CT) scan showed 1.8 cm enhancing lesion in the middle third of renal allograft (Figure 1(a)). The patient was diagnosed with acute gastroenteritis and mass suspicious for malignancy in the renal allograft. He was treated with cefazolin and gentamycin, which resolved the gastroenteritis. Later, the patient was admitted electively and underwent embolization of the renal artery of the renal allograft (Figure 1(b)) and graft nephrectomy.

Gross examination of the graft nephrectomy specimen showed well-defined, solitary, round, mahogany corticomedullary tumour in the mid zone of the renal allograft, measuring 1.6 cm in maximum dimension. No tumour central scar, haemorrhage, or necrosis was seen. The tumour was confined to the kidney and showed no extracapsular extension. Sections from the tumour were fixed in 10% buffered formalin, paraffin-embedded, sectioned at 5 *μ*m, mounted on coated glass-slides, and stained by routine haematoxylin and eosin stain (H&E). A panel of diagnostic immunohistochemical (IHC) stains was performed (Table 1). All immunoassays were performed according to the manufacturer's guidelines, using automated platform (Ventana Benchmark XT, Tucson, AZ, USA) and heat antigen retrieval by ultracell condition solution PH 8.4. Ultraview universal DAB detection kit was used for reaction visualization. Proper positive and negative controls were utilized for all IHC stains.

**Figure 2:**
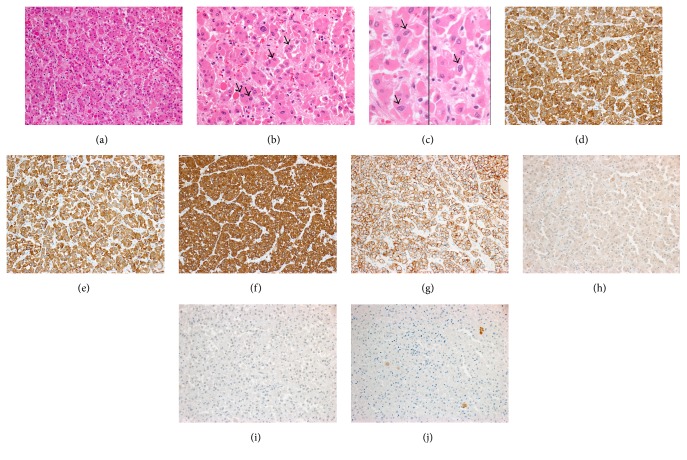
(a) Histopathological examination of the renal allograft tumour showed eosinophilic chromophobe renal cell carcinoma. The tumour consisted of medium to large round and polygonal neoplastic cells arranged in variably sized nests and cords and poorly formed acini [H&E ×200]. (b) Tumour cells exhibited hyperchromatic nuclei, irregular “raisinoid” nuclear membranes (arrows) and voluminous, and dense eosinophilic cytoplasm [H&E ×400]. (c) Tumour cells “raisinoid,” wrinkled nuclear membranes, and perinuclear cytoplasmic clearing (arrows) [H&E ×600]. Immunophenotypically, the tumour cells exhibited positive immune reaction to pancytokeratin (CK) (d), low-molecular weight CK (e), CK7 (diffuse and strong staining, (f)), and E-cadherin (g), while staining for renal cell carcinoma (h), CD10 (i), and CD117 (j) antibodies was negative [(d–j) ×200].

Histopathological examination showed well-circumscribed eosinophilic variant of ChRCC. The tumour consisted of medium to large, round, and polygonal neoplastic cells that exhibited mild degree of nuclear pleomorphism and arranged predominantly in variably sized nests, cords, and poorly formed acini and had hyperchromatic nuclei, indistinct nucleoli, irregular “raisinoid” or round nuclear cell membranes, abundant amount of dense eosinophilic cytoplasm, and poorly defined cell membranes (Figures 2(a)–2(c)). Scattered binucleated cells and occasional neoplastic cells with pale cytoplasm and perinuclear cytoplasmic clearing were present (Figure 2(c)). Mitotic activity was inconspicuous. No papillary growth pattern, foamy histiocytes, psammoma bodies, or background of extensive network of thin-walled “chicken-wire” vasculature was identified and no tumour necrosis was seen. There was no sarcomatoid differentiation. Lymphovascular invasion was not identified. Immunohistochemically, the neoplastic cells exhibited diffuse positive cytoplasmic staining for pancytokeratin (CK), low-molecular weight CK (CK8/18), CK7, and epithelial membrane antigen (EMA), and membranous staining for E-cadherin (Figures 2(d)–2(g)), while IHC staining for vimentin, CD10, CD117, and renal cell carcinoma (RCC) marker was negative (Figures 2(h)–2(j)). The remaining kidney showed severe segmental and global glomerular sclerosis, severe interstitial fibrosis and tubular atrophy, severe, widespread arteriolar hyalinosis, and severe chronic allograft arteriopathy.

The patient had an uneventful postoperative clinical course. On follow-up, the patient remains clinically stable on haemodialysis and without clinical or radiological evidence of locoregional recurrence or distant metastasis at 55 months after graft nephrectomy.

## 3. Discussion

Renal cell carcinoma of the native kidneys affects 1–3% of all renal transplant recipients with 5.6 years' estimated median interval time between renal transplantation and the occurrence of RCC [8]. In renal allograft, de novo RCC is rare and RCCs transmitted from donor represent only 0.02% to 0.2% of cases [9]. In a retrospective analysis, Leveridge et al. [6] reported 45 RCCs in 3,568 recipients who had renal transplant over a period of 43 years; 39 patients had RCC in the native kidney, while 8 were diagnosed with RCC in renal allograft. The majority of renal allograft tumours are diagnosed incidentally when a biopsy or radiological investigations are performed for other clinical indications [10]. The interval between transplantation and allograft tumour diagnosis ranges between 2 and 258 months (mean 56); early detected tumours most likely represent donor-derived tumours [11, 12]. In our patient, the chromophobe RCC was incidentally discovered in renal allograft 11 years after transplantation by a diagnostic workup for acute gastroenteritis and presumed to be a de novo disease. Unfortunately no genetic analysis or leukocytes antigen typing to further confirm the recipient origin of the tumour was performed.

Various histological types of renal allograft de novo RCC were reported. In most reported series of cases, papillary RCC was the most common type, followed by clear cell (conventional) RCC [6, 13]. De novo ChRCC in renal allograft is extremely rare and only five cases were found in the English literature, one in paediatric age group and the remaining ones in adults [7, 14–17].  Table 2 summarizes the clinicopathological features of reported renal allografts ChRCC.

Chromophobe RCC is uncommon distinct type of RCC that is derived from the intercalated cells in the collecting ducts and accounts of approximately 5% of all native renal neoplasms. Morphologically it is classified into two main variants: classical and eosinophilic [18]. The classical variant is the most common and characterised by alveoli and/or trabeculae of large, polygonal neoplastic cells with voluminous, pale, flocculent, reticulated cytoplasm, irregular nuclear membrane, perinuclear cytoplasmic clearing, and prominent cell membrane. The classical network of delicate, anastomosing capillaries in the background is not a usual feature of ChRCC. The eosinophilic variant of CRCC is very rare and exhibits similar nuclear features encountered in classical CRCC; however, the neoplastic cells tend to be arranged in more solid growth pattern and have dense eosinophilic, slightly granular, abundant cytoplasm and visible to indistinct cell membrane [19]. All previously reported ChRCC in renal allografts were classical variant [7, 14–17]. To the best of our knowledge, the current case represents the first eosinophilic ChRCC in renal allograft, which expands the spectrum of histomorphology of renal allograft RCC.

The histopathological differential diagnosis of eosinophilic ChRCC includes eosinophilic variant of conventional RCC, oncocytoma, and hybrid oncocytic/chromophobe tumour/renal cell carcinoma (HOCT). The latter two entities can be very difficult to distinguish even for an experienced urological pathologist. Several histological, histochemical, and immunohistochemical features distinguish ChRCC from other mimicking entities. In the current case the absence of typical tumour vascular component and the immunophenotypical features of the tumour easily excluded eosinophilic variant of conventional RCC, and the presence of readily identified irregular, raisinoid nuclear contour and cytoplasmic perinuclear clearing and strong diffuse immunohistochemical positivity for CK7 favour ChRCC over oncocytoma (CK7 tends to be negative or only focally positive in oncocytoma). Hybrid oncocytoma/chromophobe tumour/renal cell carcinoma is rare kidney tumour, which can be associated with renal oncocytosis and Birt-Hogg-Dube syndrome; rare sporadic HOCT were also described. These tumours exhibit histopathological features of both renal oncocytoma and ChRCC and can be extremely difficult to classify [20]. Although some neoplastic cells with round nuclear contour were seen in our case, noticeable numbers of cells with raisinoid nuclear membrane and the strong diffuse cytoplasmic (rather than focal or peripherally enhanced positivity) made us lean toward the diagnosis of eosinophilic variant of ChRCC over HOCT [21]. It is worth mentioning that overreliance on CK7 as main discriminator between eosinophilic variant of ChRCC and HOCT is not recommended as cases with strong diffuse IHC reaction can be also encountered in HOCT and cases demonstrating classic cytological features of ChRCC can be negative [22]. Thus, favoured diagnosis is a subjective matter and ultimately depends on the conventional light microscopic examination of H&E stained slides in many cases. Molecular genetics techniques such as fluorescence in situ hybridization (FISH) demonstrating variable monosomy for chromosome 1, 2, 6, 10, 13, 17, 21, or Y can be very valuable in diagnosing and help in distinguishing ChRCC from other mimickers, particularly oncocytoma, which shows no chromosomal loss. However an overlapping numerical chromosomal changes with HOCT are well documented as these tumours can exhibit mono- and polysomies of chromosomes 1, 2, 6, 9, 10, 13, 17, 21, and 22 [21]. Unfortunately molecular genetics studies were not performed on our case.

In general, ChRCC tends to be low-grade and has better prognosis compared to conventional (clear) RCC with a mortality rate < 10% [19]. Large tumour size and presence of tumour necrosis and sarcomatoid differentiation are associated with worse prognosis. Our patient had well-circumscribed tumour that was confined to the renal allograft and showed no evidence of necrosis or sarcomatoid change.

Renal cell carcinoma can be silent and has insidious biological behavior, which result in a large tumour size. Similar to the current case, all reported ChRCC were discovered incidentally by radiological investigations for other clinical indications [7, 14] or as part of routine checkup or surveillance [15, 16]. This highlights the importance of the clinical relevance of radiological graft screening in kidney transplant recipients, which allow early detection of the tumour, provide wider range of treatment options, and result in excellent clinical outcome. Ploussard et al. [15] follow up a cohort of 2396 renal transplant recipients by annual US and reported 17 RCC arising in renal allograft, of which all were staged T1N0M0 and only one patient had a tumour > 4 cm.

Renal allograft tumours can be treated by different treatment modalities that include graft nephrectomy, nephron-sparing surgery, cryoablation, and radiofrequency ablation [23]. Allograft nephrectomy is indicated in tumours larger than 4 cm, in tumours located in the mid zone of the graft, in a recurrent disease, or in tumours arising in a nonfunctional graft. Nephron-sparing surgery or other nephron-sparing therapeutic options should be considered in small tumours with low-risk of local recurrence, especially in functioning renal allograft [15]. In our case, the renal allograft tumour was surgically treated by graft nephrectomy because the graft was nonfunctional and because of anatomical location of the tumour.

In summary, ChRCC is extremely rare in renal allograft. We described a case of incidentally discovered, de novo eosinophilic variant of ChRCC, originating in a nonfunctional renal allograft 11 years after transplantation, which was treated by graft nephrectomy. This is the first case of eosinophilic ChRCC, which expands the spectrum of morphological variants of renal allograft RCC. Early detection of renal allograft tumours by annual radiological screening should be implemented regardless of the graft functionality throughout recipient's lifetime.

## Figures and Tables

**Table 1:** Primary antibodies used for IHC, their clone, dilution, and source.

Antibody	Clone	Dilution	Source
Pan CK	AE1/AE3	1/100	Cell Marque, CA, USA
CK8/18	TSIFB5	RTU	Ventana, AZ, USA
CK7	OV-TL12/30	1 : 50	Cell Marque
EMA	E29	1 : 50	Cell Marque
E-Cadherin	NCH-38	1 : 50	Cell Marque
RCC	SPM314	1 : 20	Dako, Denmark
Vimentin	V9	1 : 300	Dako
CD10	56C6	RTU	Ventana
CD117 (C-KIT)	YR145	1 : 400	Cell Marque

CK, cytokeratin; RTU, Ready to Use; EMA, epithelial membrane antigen; RCC, renal cell carcinoma.

**Table 2:** Clinicopathological features of reported chromophobe renal cell carcinoma in renal allograft.

Reference	Age at RCC diagnosis (yr)/sex	Primary kidney disease	Interval before RCC diagnosis (yr)	Treatment	Tumour location	Tumour size (cm) & histological variant	Clinical follow-up
Greco et al. [14], 2005	13.5/M	Juvenile nephronophthisis	5	Graft nephrectomy	Near the hilum	2.1, classical	N/A
Ploussard et al. [15], 2012^†^	58/M	Polycystic kidney	8	Graft nephrectomy	Upper pole	0.5, classical	Alive, NED at 96 mo
Ajabnoor et al. [16], 2014	52/F	N/A	15	Graft nephrectomy	Mid renal	8, classical	N/A
Troxell and Higgins [17], 2016	27/M	N/A	9	Partial graft nephrectomy	Unknown	3.5, classical	Graft failed at 5 yr; NED at 6 yr, died of unrelated cause
Althaf et al. [7], 2016	20/F	Unknown	5	Graft nephrectomy	Renal pelvis	3.5, classical	N/A
Current case	31/M	IC-MPGN	11	Graft nephrectomy	Mid renal	1.6, eosinophilic	Alive, NED at 55 mo

^†^Chromophobe RCC was combined with papillary RCC measuring 4 cm and conventional (clear) cell RCC measuring 0.5 cm. RCC, renal cell carcinoma; yr, year; cm, centimeter; N/A, not available; NED, no evidence of disease; mo, months; IC-MPGN, immune-complex mediated membranoproliferative glomerulonephritis.
